# The tale of a transposition error

**DOI:** 10.1002/hem3.70415

**Published:** 2026-07-20

**Authors:** Stephen P. Hibbs

**Affiliations:** ^1^ Wolfson Institute of Population Health Queen Mary University of London London UK

The fear hit me with a wave of nausea. I was preparing to give a talk for a group of students about a research project I had co‐led, which highlighted how cancer clinical trials and therapies structurally discriminate against people with the Duffy null variant, common in people of African and Middle Eastern ancestry. By accounting for Duffy‐related variation in absolute neutrophil count, clinical trial eligibility criteria could exclude fewer people, and prescription guidance could result in fewer unnecessary dose interruptions or reductions. Our paper had been published 2 months earlier.^1^

I returned to the study datasheet to pick out a couple of examples of trials to show to the students. This should have been a straightforward task. Instead, I came across something surprising—a trial that one column of my datasheet categorized as “UK‐ or US‐based” only listed locations in China. What had gone on?

With a knot in my stomach, I gave a half‐hearted talk to the students and went home. Lying awake, my thoughts swirled. If the location of this trial was miscategorized, was this the case for others too? Or even worse—were other fields of the dataset wrong? How deep did the problem go?

I felt the significance of this paper. Our findings illuminated a significant aspect of cancer health inequity. We planned to use these results to influence clinical practice, and an accompanying discussion paper with policy recommendations was in press.^2^ Would finding and reporting a data error jeopardize the core message we were trying to communicate, delaying progress in the real world? And how would others in the research team respond to any error I had made? These were people I respected, but within our trans‐Atlantic collaboration, I knew few of them personally.

As these questions swirled, another thought skulked across my mind—perhaps I could allow myself to forget what I saw? After all, had I not been preparing this teaching session, I might never have known that there were any problems in the data. It seemed unlikely that anyone else would ever notice.

I slept restlessly, but by the morning, I decided that I could not “unsee” this. I needed to look back at the dataset, understand the extent of the problem, and follow through with the consequences, however exposing they might be. I sat down at my desk with a strong cup of coffee, took a deep breath, and opened my computer. Within a few minutes, the problem was clear—I had made a transposition error where all location assignments were mismatched by two rows, resulting in the location status of each trial being taken from an unrelated study.

Looking through the dataset, I was relieved to find that other elements of data were not affected by my transposition error. Location was used for a sensitivity analysis rather than our main results. The consequences were real, but limited: the sensitivity analysis and its corresponding supplementary figure were incorrect after reanalysis with the corrected data, though their interpretation remained the same.

Now that I understood where we stood, I wrote an email to the senior author to describe the error and the ways in which it affected our paper, asking his advice on next steps. Within a few hours, he replied, opening with an affirmation that I was doing the right thing by being forthright about the error. I was grateful for his words at this time. He explained the next steps—we would need to issue a correction, write a letter of explanation, and correct the manuscript and supplementary information. We worked together through the day to recheck the reanalysis and the rest of the data and set to work on the correction. The correction was issued, and as far as I can tell, has not impeded the contribution or impact of the study as I had feared it might.

In some ways, this is not a particularly dramatic story. But despite working in various research teams for around 15 years at this point, I had not heard anyone describe an experience of noticing an error in their data after publication, or of the experience of issuing a correction. Perhaps I had not been in the right rooms, or perhaps there is some taboo around errors, corrections, and retractions. My own fear was amplified by this taboo: I conflated my avoidable research error with stories I knew of willful research malpractice, “gotcha” stories about rogue researchers being exposed. During the correction process, I found a helpful editorial from the journal group, delineating the difference between accidental errors—even if they were substantive or pervasive errors—and scientific misconduct.^3^ This helped me understand where I stood.

In contrast, I remember each of the occasions I first made a clinical error, was involved in a serious incident, or was named in a complaint from a patient. Although my heart sank with the same dread, I had the benefit of having heard others speak about their experiences—I knew and respected colleagues who had told me how they had dealt with such incidents.^4^ A mentor once said to me “after a certain amount of time working in medicine, if you've not been involved in an incident or complaint, you probably aren't doing enough work.”

The acceptability of admitting error will vary substantially across different research and clinical environments, but I have experienced many examples of transparency within my clinical training. One example is transfusion: haemovigilance systems have normalized reporting errors to the extent that there is concern where *not enough* incidents are reported, rather than too many.^5^ The road to transparency in clinical error was hard fought and often led by patient‐safety campaigners. Perhaps some research cultures would benefit from a similar shift.

I now look back on my transposition error with a sense of relief. I do not think issuing the correction changed anything about the meaning, interpretation, or impact of the study, but it did change me. Had I decided to “unsee,” I would still be carrying a residue of that fear and guilt, watching my back a little, never quite enjoying the research contribution I had made, and trusting myself less to be transparent in the future. I recognize that it is far easier for me to have owned this error than for researchers in more precarious situations: holding insecure contracts, operating in brutal research cultures, or subject to forms of minoritization. I did not fear that owning this mistake would be used against me viciously; this assumption cannot be taken for granted by others.

My transposition error was avoidable. It was embarrassing. I have learned some practical lessons that reduce the risk of making this same mistake in the future. However, it is highly likely, perhaps inevitable, that I will publish further errors in my research career. It is possible that some will change the whole interpretation of a study—perhaps requiring a full retraction. When the day comes that I spot my next mistake, I hope this experience will have helped me prepare to hold my hands up, to own the consequences, and to move on without bearing a troubled conscience. In writing this, I hope someone else reading this finds their own errors feel a little less heavy, too.

## AUTHOR CONTRIBUTIONS

**Stephen P. Hibbs**: Conceptualization; writing—original draft; writing—review and editing.

## CONFLICT OF INTEREST STATEMENT

The author declares no conflicts of interest.

## FUNDING

S.P.H. is supported by a HARP doctoral research fellowship, funded by the Wellcome Trust (Grant number 223500/Z/21/Z).

## Data Availability

Data sharing not applicable to this article as no datasets were generated or analyzed during the current study.
